# One shot NEPA plus dexamethasone to prevent multiple-day chemotherapy in sarcoma patients

**DOI:** 10.1007/s00520-019-4645-3

**Published:** 2019-02-14

**Authors:** Giuseppe Badalamenti, Lorena Incorvaia, Carlo Messina, Emmanuela Musso, Alessandra Casarin, Maria Rita Ricciardi, Ida De Luca, Viviana Bazan, Antonio Russo

**Affiliations:** 1 Department of Surgical, Oncological and Oral Sciences, Section of Medical Oncology, University of Palermo, Palermo, Italy; 2 Medical Oncology Unit, Santa Chiara Hospital, Trento, Italy; 3 Department of Experimental Biomedicine and Clinical Neurosciences, School of Medicine, University of Palermo, Palermo, Italy

**Keywords:** Netupitant, Palonosetron, Sarcoma, Multiple-day, CINV

## Abstract

**Purpose:**

Chemotherapy-induced nausea and vomiting (CINV) is one of the most feared and disturbing adverse events of cancer treatment associated with decreased adherence to effective chemotherapy regimens. For high-risk soft tissue sarcoma patients, receiving multiple-day chemotherapy (MD-CT), antiemetic guidelines recommend a combination of an NK_1_ receptor antagonist (NK_1_-RA), a 5-HT_3_ receptor antagonist (5HT_3_-RA), and dexamethasone on each day of the antineoplastic treatment. NEPA is the first oral fixed-dose combination of a highly selective NK_1_-RA, netupitant, and second-generation 5HT_3_-RA, palonosetron. So far, no data has been published in literature about the efficacy of a single dose of NEPA in MD-CT.

**Methods:**

We performed a prospective, non-comparative study to assess the efficacy of one shot of NEPA plus dexamethasone in sarcoma patients receiving MD-CT. The primary efficacy endpoint was a complete response (CR: no emesis, no rescue medication) during the overall phase (0–120 h) in cycle 1. The main secondary endpoints were CR during the overall phase of cycles 2 and 3.

**Results:**

The primary endpoint was reached in 88.9% of patients. Cycles 2 and 3 overall CR rates were 88.9% and 82.4%, respectively. The antiemetic regimen was well tolerated.

**Conclusions:**

This pilot study showed the benefit of one shot of NEPA to prevent CINV in sarcoma patients receiving MD-chemotherapy.

## Introduction

Chemotherapy is still the mainstay treatment of several solid tumors, and chemotherapy side effects (CSEs) are often responsible for quality of life (QoL) deterioration impairing patients’ ability to manage daily activities [1]. In a recent study, Lorusso et al. highlighted that chemotherapy-induced nausea and vomiting (CINV) is one of the most feared adverse event**s** before starting chemotherapy and is still the most commonly experienced during treatment. Prophylaxis for CINV is still an unmet medical need in cancer treatment, and antiemetic strategies should be improved in the future [2]. CINV risk factors are well defined for both chemotherapy regimens and patients: type, dose and chemotherapy schedule, female gender, young age (< 55 years), non-users of alcohol, previous nausea and vomiting due to cancer treatment or pregnancy, anxiety, and motion sickness [3].

International guidelines provide recommendations for antiemetic prophylaxis according to the emetogenic potential of chemotherapy [4–6]. High and moderate emetogenic chemotherapies need a multi-drug approach with a combination of 5-HT_3_ and NK_1_ receptor antagonists. Chemotherapy regimens for soft tissue sarcoma patients are often delivered for multiple days, often resulting in poor management of CINV due to the daily infusion of chemotherapy. Antiemetic guidelines in patients receiving multiple-day chemotherapy (MD-CT) recommend the use of antiemetic drugs before treatment that are appropriate for the emetic risk of the antineoplastic agent administered on each day of the antineoplastic treatment and for 2 days after completion of the antineoplastic regimen. In high-risk patients receiving MD-CT, a three-drug combination of an NK_1_ receptor antagonist (NK_1_-RA), a 5-HT_3_ receptor antagonist (5HT_3_-RA), and dexamethasone should be considered. The efficacy of different antiemetic triplet regimens has been evaluated in sarcoma patients receiving MD-CT: aprepitant plus dexamethasone in combination with one shot of palonosetron or multiple days of granisetron. No significant differences have been detected between the two antiemetic regimens, and CINV control has been considered insufficient, with less than 50% of patients having been controlled [7]. The combination of netupitant 300 mg plus palonosetron 0.50 mg (NEPA) has recently been approved as a prophylactic antiemetic strategy for patients treated with chemotherapy. Netupitant, the NK_1_-RA component of NEPA, is a new highly selective NK_1_-RA that can saturate NK_1_ receptors up to 90% and has a longer half-life (96 h) than aprepitant (9–13 h) [8]. The rationale for the combination of the two active principles of NEPA is based on their unique and complementary action on the NK_1_ receptor [8]. No data has been published in literature about the efficacy of a single dose of NEPA in MD-CT, neither in the oncology nor in the hematology setting.

Therefore, we carried out a pilot study to assess the efficacy of one shot of NEPA plus dexamethasone in sarcoma patients receiving MD-CT.

## Methods

This prospective, non-comparative study was conducted in the Oncology Department of Palermo University, Italy. The study was approved by the local ethics committee, and all patients signed the informed consent form. Eligible patients were 18 years old or older, with a diagnosis of soft tissue tumors and were scheduled to receive a MD-CT of epirubicin (EPI) 35 mg/m^2^ days 1–3 and ifosfamide (IFO) 3000 mg/m^2^ days 1–3 every 21 days. Other eligibility criteria were ECOG 0–2, adequate bone marrow function, hepatic and renal function, and the willingness and ability of patients to complete a diary. The main exclusion criteria were the presence of vomiting or nausea before chemotherapy administration and hypersensitivity to palonosetron or netupitant.

For antiemetic prophylaxis, all patients received a single dose of NEPA on day 1 only and dexamethasone 12 mg on days 1, 2, and 3. A dose escalation of dexamethasone was done, 4 mg/bid on days 4, 5, and 6 and 2 mg/bid on days 7, 8, and 9. Metoclopramide has been prescribed as rescue medication.

Patients kept a self-assessed diary, from day 1 to day 7 to assess nausea (Likert scale: none, mild/moderate, or severe) and record episodes of vomiting. A 10-point visual analogue scale was completed by patients at the end of the 7-day assessment. In the morning of day 8, the patients returned their diaries, and all the data entered were discussed with the physician.

The primary endpoint of the study was to evaluate the complete response (CR) rate during the first chemotherapy cycle of EPI-IFO, during the overall phase (0–120 h after chemotherapy administration). CR is defined as no vomiting and no use of rescue medication. Other endpoints were CR during the acute (0–24 h) and delayed (25–120 h) phase, no vomiting and no nausea during the acute, delayed, and overall phase of the first and subsequent cycles (maximum 3 cycles).

Treatment safety was evaluated during all chemotherapy cycles, and all adverse events were recorded and graded according to the common terminology criteria for adverse events (CTCAE) described by the National Cancer Institute, version 4.0. (http://ctep.cancer.gov/forms/CTCAEv4.pdf).

Demographic data and patient characteristics were examined. The percentage of patients with CR and no vomiting or nausea for the acute, delayed, and overall period was calculated.

## Results

Between January 2017 and June 2017, 18 patients affected by sarcoma were enrolled. Fifty percent were male; the median age was 53 (range 25–80), and patients received chemotherapy for metastatic (8/18), neoadjuvant adjuvant (8/18), or adjuvant (2/18) intent. Seventeen patients completed the three planned EPI-IFO cycles, while one patient did not undergo the third cycle of treatment, due to disease progression. Four patients had received chemotherapy treatment previously, while 14 (77.8%) patients were naïve. Among the pre-treated patients, one patient received chemotherapy for Hodgkin Lymphoma in 2001, one patient for breast cancer in 2010, and 2 patients received chemotherapy for sarcoma, but they did not experience nausea and vomiting during the treatment.

**Fig. 1:**
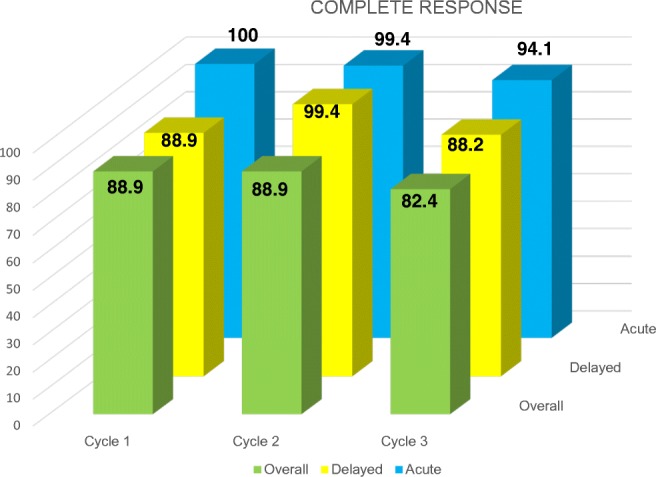
Patients achieving a complete response (no vomiting and no use of rescue medication) during the acute (0–24), delayed (25–120), and overall phase (0–120 h) of the three planned cycles

**Fig. 2:**
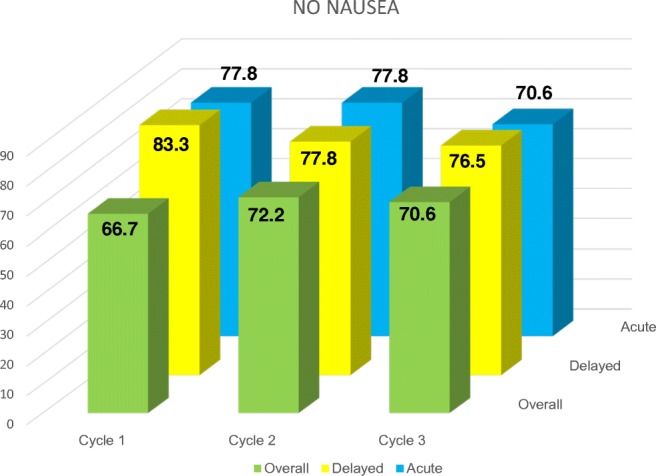
Percentage of patients reporting no nausea episodes during the acute (0–24), delayed (25–120), and overall phase (0–120 h) of the three planned chemotherapy cycles

The primary endpoint was 88.9% of CR during the overall phase. The no vomiting rate and CR rate are identical. None of the patients required rescue medication at home during the 7 days of assessment. Figures 1 and 2 report the results of CR-no vomiting and no nausea, respectively. All patients were compliant to dexamethasone during the delayed phase.

No grade 3 or 4 toxicity was detected during the entire study period. Patients experienced the following grades 1 and 2 adverse events: headache 16.6% (3/18), constipation 38.8% (7/18), hiccups 22% (4/18), anorexia 5.5% (17/18), insomnia 27.7% (5/18), and heartburn 22.2% (4/18).

No neurotoxicity was detected in our patients.

## Discussion

Antiemetic guidelines suggest a multi-therapy approach with a combination of 5-HT_3_ and NK_1_ receptor antagonists for patients receiving MD-CT [4–6]. It is well known that CINV control could be improved by following antiemetic guidelines. However, published studies have shown that it is often suboptimal [9, 10]. A multi-therapy approach to manage nausea and vomiting during all 5 days of the scheme could make patient adherence more difficult, because it requires self-administration of the medication at home. A simply guideline-based antiemetic prophylaxis is warranted in oncology patients to enhance adherence and, therefore, efficacy [11], and NEPA appears to be an advancement, particularly in the setting of MD-CT [7].

To our knowledge, this is the first study evaluating the efficacy and safety of one shot antiemetic drug of NEPA plus dexamethasone to prevent CINV prophylaxis in the MD-CT. In our study design, we assessed whether a single dose of NEPA could cover the whole 5-day risk period of CINV (3 days of chemotherapy plus 2 days after).

A single dose of NEPA plus dexamethasone covers all 7 days after starting chemotherapy both for vomiting and nausea, reaching a high level of control during the entire study period (acute, delayed, and overall) and for all three cycles. Our efficacy results are higher, for both nausea and vomiting control, with respect to previous reports combining NK_1_-RA and 5HT_3_-RA [7]. Kimura and collaborators assessed the efficacy of aprepitant plus granisetron or palonosetron and this three-drug combination was not sufficient to control CINV in sarcoma patients receiving MD-CT. This study also demonstrated that consecutive-day granisetron was not inferior to single-shot palonosetron for treating CINV.

The safety profile of NEPA in our study is of particular interest, because it is well known that NK_1_-RA interacts with cytochrome: netupitant is an inhibitor of the CPY3A4, and aprepitant and fosaprepitant are both inhibitors and inducers of CPY3A4, while rolapitant is an inhibitor of CPY2D6 and it interacts with PgP (see Table 1) [12–15]. In our study, the safety profile of NEPA with respect to possible clinical interaction is confirmed [16–18]. Additionally, in our patients, we did not detect, as expected [19], an increase in ifosfamide neurotoxicity. This adverse event is reported for aprepitant/fosaprepitant, since it is a CYP3A4 inducer.

**Table 1:** Clinically relevant cytochrome interactions of NK_1_-RA

Cytochrome	Netupitant [15]	Aprepitant [12–19]	Rolapitant [14]
CYP3A4 inhibition	Dex, Ketoconazole	Dex, CTX^†^	No
CYP3A4 inducer	Rifampicin	CTX^‡^	No
CYP2C9 inducer	No	Warfarin, oral contraception	No
CYP2D6 inhibition	No	No	Drugs^§^
P-gP	No	No	CTX^¶^

^†^Cyclophosphamide, bosutinib, cabazitaxel

^‡^Ifosfamide, etoposide, vinorelbine, docetaxel, paclitaxel

^§^Anti-arrhythmics, anti-depressants, antipsychotics, beta-blockers, analgesic

^¶^Irinotecan, doxorubicin, methotrexate, bendamustine, rosuvastatin

*CTX*, chemotherapy; *Dex*, dexamethasone

In the setting of MD-CT, the choice of one-shot NEPA schedule would allow the simplification of therapy by decreasing the number of individual dose units to be taken by the patient, simplifying therapy, and improving patient compliance. The correct timing and administered dose of the drugs should be considered in order to optimize the prophylactic treatment [20].

The limitation of our study is the small sample of patients, but sarcoma tumor is a rare disease. Our study results should be confirmed in future studies. Moreover, further studies are needed to assess whether NEPA could allow a dexamethasone sparing approach for antiemetic prophylaxis due to its long half-life and to the synergy of the two active drugs on the NK_1_-receptor [21].

## Conclusions

Nowadays, although advances have been made in the management of nausea and vomiting, CINV remains one of the most dreaded side effects of chemotherapy. When poorly controlled, CINV can negatively impact the patient’s ability to tolerate chemotherapy and can affect their quality of life.

It has been demonstrated that adherence to antiemetic guidelines guarantees more effective control of CINV. In our study, one-shot NEPA administration appears to be an advance, particularly with its simplicity of administering guideline-based antiemetic prophylaxis.
